# Highly elastic 3D-printed gelatin/HA/placental-extract scaffolds for bone tissue engineering

**DOI:** 10.7150/thno.73146

**Published:** 2022-05-13

**Authors:** JiUn Lee, Dongyun Kim, Chul Ho Jang, Geun Hyung Kim

**Affiliations:** 1 Department of Biomechatronic Engineering, College of Biotechnology and Bioengineering, Sungkyunkwan University (SKKU), 16419, Suwon, Republic of Korea; 2 Department of Otolaryngology, Chonnam National University Medical School, Gwangju 61186, Republic of Korea; 3 Biomedical Institute for Convergence at SKKU (BICS), Sungkyunkwan University, Suwon 16419, Republic of Korea

**Keywords:** gelatin, placental-extracts, scaffold, bone, tissue engineering

## Abstract

Bioengineering scaffolds have been improved to achieve efficient regeneration of various damaged tissues. In this study, we attempted to fabricate mechanically and biologically activated 3D printed scaffold in which porous gelatin/hydroxyapatite (G/H) as a matrix material provided outstanding mechanical properties with recoverable behavior, and human placental extracts (hPE) embedded in the scaffold were used as bioactive components.

**Methods:** Various cell types (human adipose-derived stem cells; hASCs, pre-osteoblast; MC3T3-E1, human endothelial cell line; EA.hy926, and human dermal fibroblast; hDFs) were used to assess the effect of the hPE on cellular responses. High weight fraction (~ 70 wt%) of hydroxyapatite (HA) in a gelatin solution supplemented with glycerol was used for the G/H scaffold fabrication, and the scaffolds were immersed in hPE for the embedding (G/H/hPE scaffold). The osteogenic abilities of the scaffolds were investigated in cultured cells (hASCs) assaying for ALP activity and expression of osteogenic genes. For the *in vivo* test, the G/H and G/H/hPE scaffolds were implanted in the rat mastoid obliteration model.

**Results:** The G/H/hPE scaffold presented unique elastic recoverable properties, which are important for efficient usage of implantable scaffolds. The effects of G/H and G/H/hPE scaffold on various *in vitro* cell-activities including non-toxicity, biocompatibility, and cell proliferation were investigated. The *in vitro* results indicated that proliferation (G/H = 351.1 ± 13.3%, G/H/hPE = 430.9 ± 8.7% at day 14) and expression of osteogenic markers (*ALP*: 3.4-fold, *Runx2*: 3.9-fold, *BMP2*: 1.7-fold, *OPN*: 2.4-fold, and *OCN*: 4.8-fold at day 21) of hASCs grown in the G/H/hPE scaffold were significantly enhanced compared with that in cells grown in the G/H scaffold. In addition, bone formation was also observed in an *in vivo* model using rat mastoid obliteration.

**Conclusion:** *In vitro* and *in vivo* results suggested that the G/H/hPE scaffold is a potential candidate for use in bone tissue engineering.

## Introduction

Various bone grafts such as autografts and allografts have been employed for the repair and regeneration of bone defects. Autografts are gold standard for treating bone defects because of their non-immunogenic property and outstanding histocompatibility 1. However, to obtain autografts, additional operations are required to harvest the patient's own bone tissue 2. Allografts, alternative to autografts, are bone tissue extracted from cadavers. These bone tissues also have histocompatible properties, and their shape can be easily processed into several forms that can be easily applied to the area of defective bone tissues 2. However, allografts are not free from the potential risks of immunoreactions and disease transmission 1. To overcome the problems of grafts, such as limited supply, donor site morbidity, immune response, and disease transmission, tissue-engineering strategies have been investigated as potential treatment choices. To successfully achieve this goal, various important elements should be prepared based on the strategies for mimicking the bone tissues or decellularized extracellular matrix of bone: a biocompatible scaffold mimicking natural bone tissues, osteogenic cells, biophysical and biochemical cues to help induce the cells to the phenotypically necessary cell type, and finally adequate vascularization 3.

In general, various biofabrication methods such as 3D printing 4, electrospinning 5, and cell spheroid fabrication processes 6 have been used to fabricate tissue engineering scaffolds. Among these techniques, 3D printing is considered an outstanding method because it is a cost-effective and versatile method, which is appropriate for obtaining complex 3D structures with microscale mesh struts 7. Using the 3D printing method, the 3D specific architecture of the artificial bone matrix can be easily achieved. The controllability of pore geometries and internal pore structures can also provide sufficient spatial space for forming new blood vessels that are required to regenerate volumetric bone tissue and metabolic activities between the construct and the environment 8. The highly personalizable strengths of the 3D printing can be adapted to bone defects with various types of structures, such as fully interconnected pore structure 9, hierarchical structure 10, fibrous structure 11, and cell- or growth factors- laden structure 12 for bone tissue engineering.

Various biomaterials have been used as printing materials to fabricate tissue engineering scaffolds, and are composed of natural polysaccharide- or protein-based biopolymers, including cellulose 13, 14, chitin 15, lignin 16, hyaluronic acid, alginate 17, gelatin, collagen 18, fibrin, and silk fibroin 19. Gelatin derived from collagen hydrolysis has been widely used in various tissue engineering applications, drug carriers, scaffolds, and wound dressings because of its good biocompatibility, low immunogenicity, and appropriate biodegradability 20. Furthermore, due to the collagen-binding proteins of gelatin, various cellular activities including cell adhesion, migration, and even growth can be promoted 20. Although meaningful bioactive properties have been revealed, the poor mechanical nature of gelatin has been an issue in the fabrication of scaffolds for hard-tissue regeneration.

**Figure 1:**
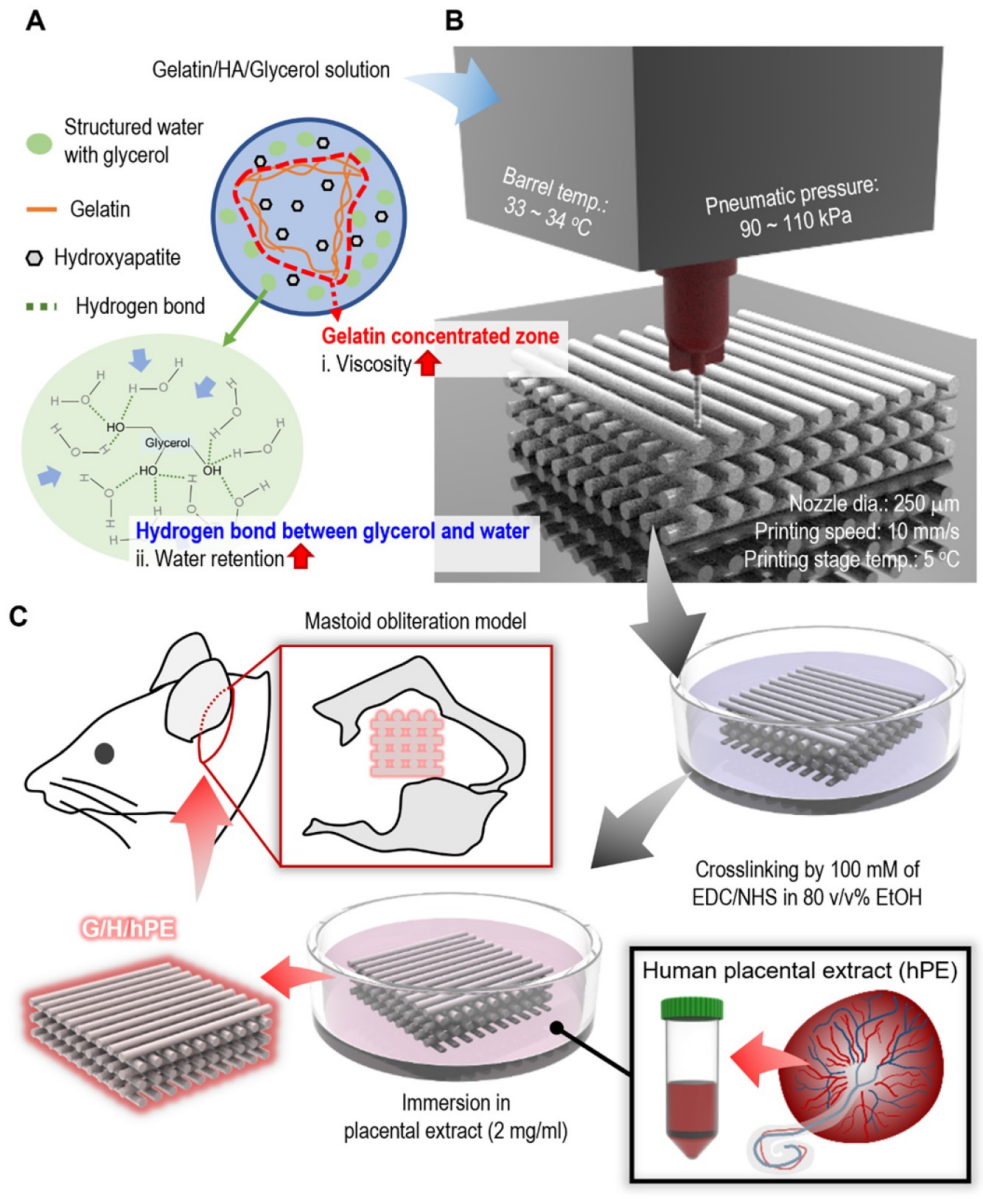
Schematics of (A) glycerol effects on gelatin/HA/glycerol ink, (B) 3D printing/crosslinking process for the gelatin/HA scaffolds, and (C) the coating of the gelatin/HA (G/H) scaffold with human placental extracts and implantation of the scaffolds into a rat mastoid obliteration model.

There are various strategies to overcome the poor mechanical properties of scaffolds, such as dual crosslinking 21, mixing fillers 22, bilayered structures 23, and structure design modification 24, etc. Among the methods, mixing fillers can be used for the improvement of not only mechanical properties, but also biological functionality such as osseointegration or osteogenesis 25. Hydroxyapatite, which represents almost 70 wt% of natural bone, has been widely used as a bioceramic filler for bone tissue engineering to mimic the natural bone 26. Therefore, we used a fixed weight fraction (~ 70 wt%) of hydroxyapatite (HA) in a gelatin solution to improve the mechanical properties of the printed gelatin-based scaffold. However, a high weight fraction of HA in a low-viscosity gelatin solution can be easily sedimented, and aggregated HA powders in the solution can impair printing quality 27. In addition, a printing solution containing high weight fraction of ceramic could be easily dried at the end of a nozzle tip during 3D printing 28, 29. For this reason, we used a sacrificial polyol, glycerol, as a printing agent to prevent the sedimentation of HA bioceramics during the printing process, as shown in Figure 1A-B. Furthermore, to enhance the bioactive properties of the fabricated gelatin/HA scaffold, human placental extracts (hPE), which contain various growth factors and cytokines inducing outstanding cellular activities such as cell growth and differentiation, were used 30. Although the hPE have been widely used for the various therapy, to the best of our knowledge, the osteogenic potential of hPE have not been clearly proved 31. To observe the osteogenic activities of the fabricated gelatin/HA/hPE (G/H/hPE) scaffolds, the expression of various osteogenic genes in human adipose stem cells (hASCs) was assessed for several culture periods. Moreover, bone formation showing the regenerative effect of the G/H/hPE scaffold was observed in an *in vivo* model using rat mastoid obliteration (Figure 1C).

## Results and Discussion

### Fabrication of gelatin/HA scaffolds

Various gelatin-based composites have been extensively used for bone tissue engineering owing to their good osteogenic cellular responses and reasonable mechanical strength 32. Generally, to mimic bone tissues, a high concentration of HA and calcium phosphate bioceremics have been used (~70 wt%) to fabricate tissue engineering scaffolds 26, 33, 34. However, sedimentation and easy aggregation of the bioceramics in hydrogel-based ink have been problems that need to overcome in scaffold fabrication using a 3D-printing process 35. In particular, the printing of gelatin-based solutions containing high concentrations of bioceramics can be more difficult than that of synthetic biopolymers because of the low viscosity, stiff sol-gel transition of gelatin, and quick drying at the nozzle tip, causing unstable printing ink extrusion.

To address the poor printing ability of the gelatin-HA (70 wt%) solution, a typical strategy is to use a temporal processing agent to control the viscosity of the printing solution 36. In this work, we adapted one of the polyols, glycerol, which is known for its water retention-deterring dehydration of the hydrogels 37, as a temporal printing supporter to obtain a stable 3D gelatin/HA construct because glycerol can form hydrogen bonds with water, resulting in a significant increase in the solution viscosity (Figure 1A) 38.

**Figure 2:**
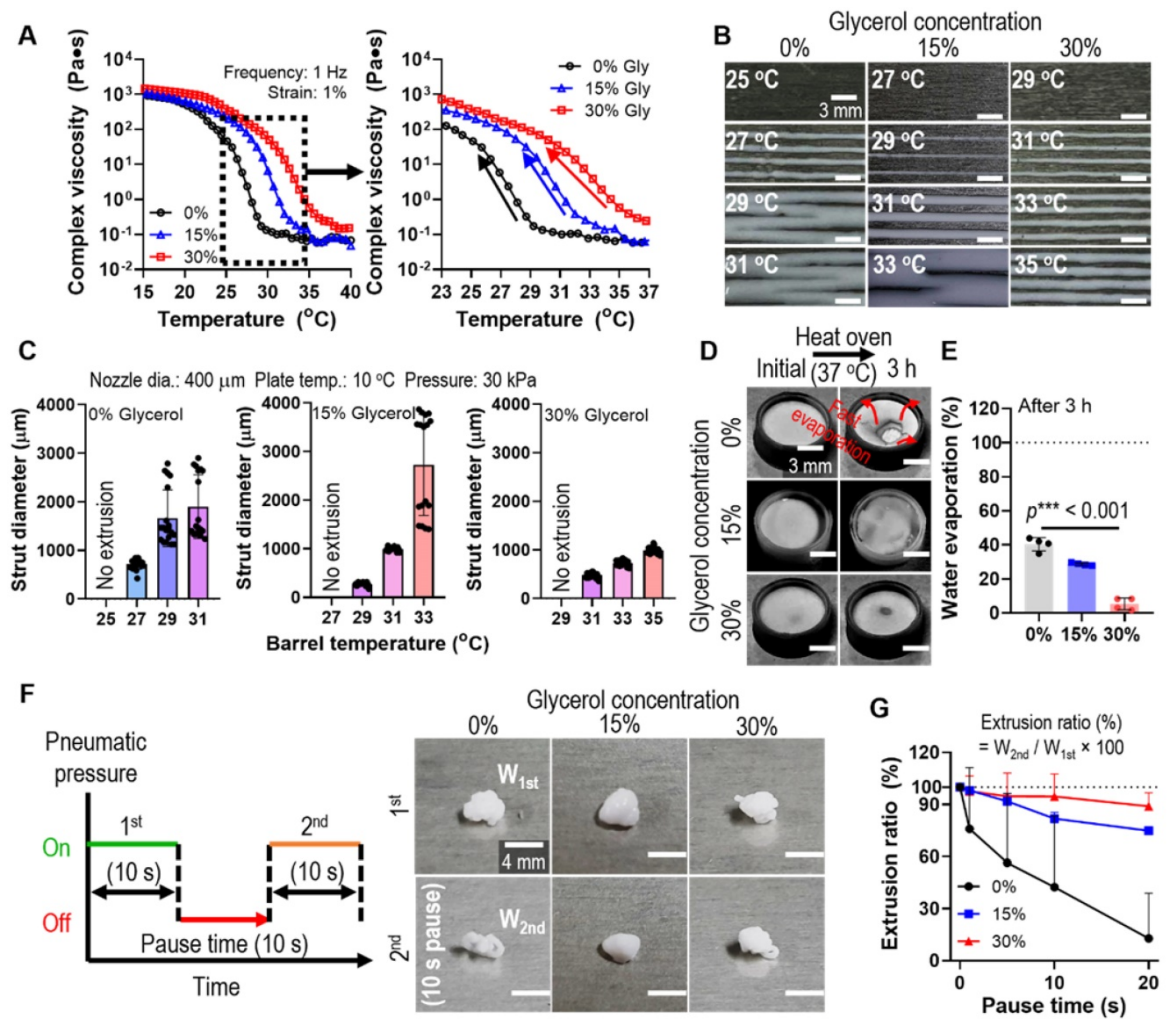
(A) Complex viscosity for temperature sweep (15 - 40 °C) of the G/H ink with various glycerol concentrations (0 ~ 30%). (B) Single line test of the G/H with and without glycerol in various printing barrel temperatures and (C) measured diameters of the single struts. (D) Optical images and (E) water evaporation (%) of each ink before and after the condition (37 °C for 3 h). (F) A schematic describing pausing time during printing and optical images showing extruded gelatin/HA ink before and after 10 s printing pause time and (G) extrusion ratio of G/H ink with and without glycerol for various printing pause times (0-20 s).

To assess the glycerol function in the printing process, we fixed the weight fraction of gelatin (10 wt%), which is generally used in tissue engineering applications, and HA powders (70 wt%). The complex viscosity (η^*^) of the gelatin/HA solution with various concentrations of glycerol (0 ~ 30 v/v%) was measured during a temperature sweep (15-40°C). As shown in Figure 2A, a much higher complex viscosity was observed in gelatin/HA with 30 v/v% glycerol (30% Gly) than in the gelatin/HA solution, and the highly stiff sol-gel transition region was slowed down. To compare the effect of the thermo-sensitivity of the G/H gel with various glycerol concentrations on the printing ability, we performed a single line test for several barrel temperatures under the printing conditions (nozzle diameter = 400 μm, working plate temperature = 10°C, and pneumatic pressure = 30 kPa) (Figure 2B). As shown in Figure 2B-C, the strut diameter of the G/H solution with 30% glycerol was much more stable and more homogeneous compared to that of the printed G/H solution with 0% and 15% glycerol, whereas G/H solution with 45% glycerol caused phase separation during the 3D printing (Figure S1).

During the 3D printing process using a high concentration of a ceramic solution, the inks were easily dried at the end of the printing nozzle causing clogging or unstable 3D printing 28, 29. We expected that the hygroscopic characteristics of glycerol can resolve the fast drying of the G/H ink. Therefore, to evaluate the water retention effect of glycerol, G/H solutions with various concentrations of glycerol were poured into a mold and incubated in an oven at 37°C for 3 h. Figure 2D shows the water retention ability using glycerol, and G/H with glycerol showed a much slower rate of water evaporation than G/H without glycerol (Figure 2E). The rapid drying of hydrogel printing ink can lead to a sudden increase in viscosity that occurs during printing, eventually blocking the microscale printing nozzle. Thus, viscosity increase (or rapid drying of printing ink) has been a major issue for hydrogel-based inks when fabricating complex 3D constructs that require a lot of time. To determine the effect of printing ink drying on extrusion ability, we measured the weights of the printed G/H with various concentrations of glycerol. Figure 2F shows the schematic of the testing method. After printing at a constant pneumatic pressure for 10 s, printing was stopped for 10 s (pausing time), and printing was continued for 10 s. The optical images showed the extruded G/H during the 1^st^ printing and the 2^nd^ printing after the pausing of time (10 s). Figure 2G shows the results of the extrusion ratio, defined as W_2nd_/W_1st_ × 100 (W_1st_: weight of G/H during the 1^st^ printing and W_2nd_: weight of G/H during the 2^nd^ printing), by changing only the pausing time. The G/H ink with 30% glycerol showed outstanding extrusion ability compared to that of G/H without glycerol and with 15% glycerol. Therefore, we chose 30% of glycerol as an optimal condition for stable 3D printing.

### Characteristics of the gelatin/HA scaffold

**Figure 3:**
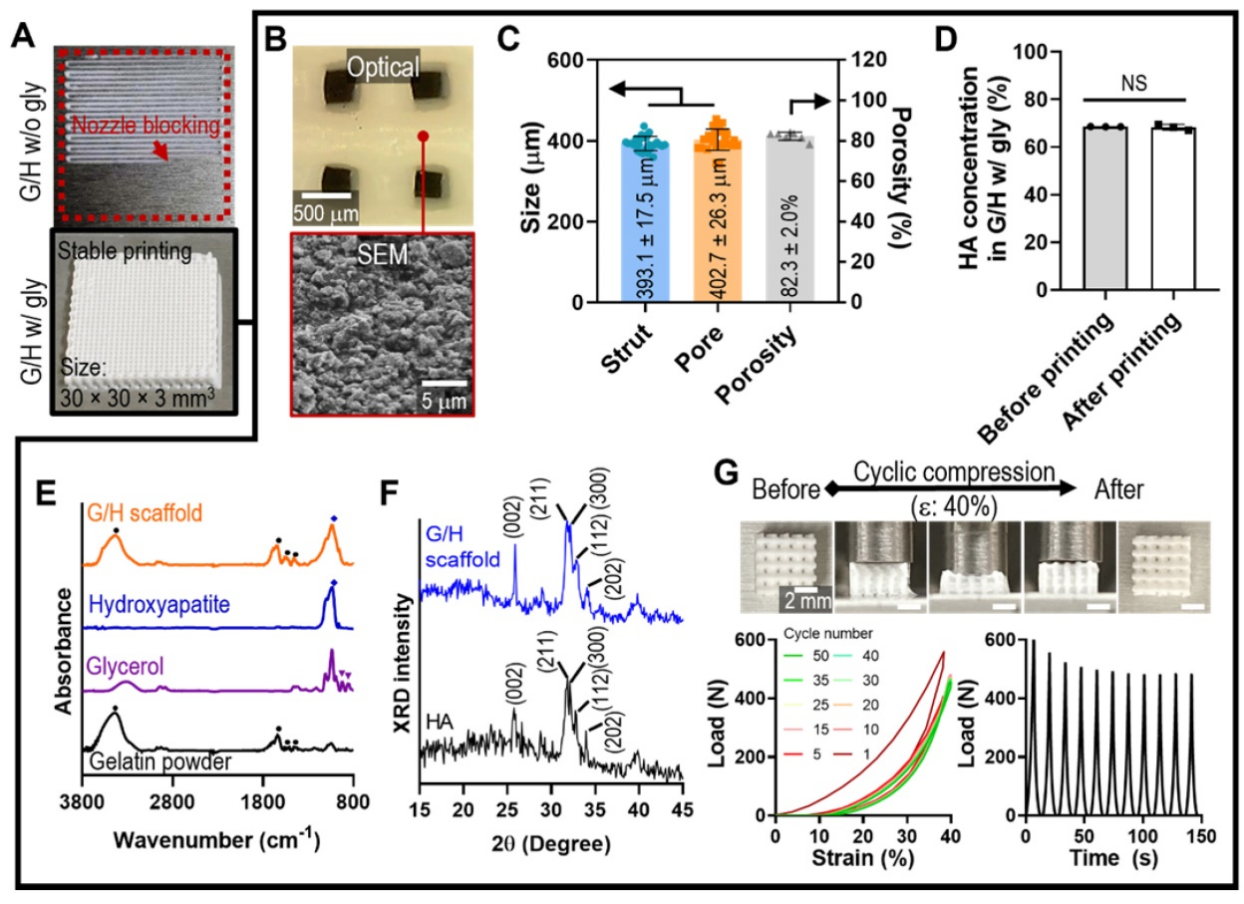
(A) Optical images showing the printability using the G/H ink with and without glycerol to obtain a cuboid structure (30 × 30 × 3 mm^3^). (B) Optical and SEM images and (C) pore geometry of the G/H scaffold fabricated using glycerol. (D) HA weight fraction in G/H scaffold before and after printing. (E) FT-IR spectra and (F) XRD results of the G/H scaffold. (G) Optical images of G/H scaffold showing shape recoverable behavior under the condition of 40% strain, 30 mm/min and compressive load-strain and load-time curves for several cycles. (NS = not significant, ^*^p < 0.05, ^**^p < 0.005, ^***^p < 0.001)

As a scaffold for bone tissue engineering, we designed a cuboid structure (30 × 30 × 3 mm^3^) with appropriate pore geometry. As shown in Figure 3A, printing with ink containing G/H with 30% glycerol resulted in the formation of a stable mesh structure; however, printing failed when ink containing G/H without glycerol was used because of nozzle blocking, which was expected because of the nozzle blocking phenomenon, which was expected from the results of Figure 2G.

Generally, pore geometry in a biomedical scaffold is an important factor for bone regeneration in terms of cell infiltration and vascularization 39, 40. Especially, 300 - 400 μm of pore structure allows the blood vessel ingrowth, endochondral ossification, and bone maturation 41, 42. Therefore, we set to produce a mesh scaffold with 400 μm pore and strut size, using the following printing conditions: barrel temp.: 33-34 °C, pneumatic pressure: 90-110 kPa, nozzle diameter: 250 μm, printing speed: 10 mm/s, and working stage temperature: 5 °C. After printing, the mesh structure was cross-linked with 100 mM EDC/NHS for 24 h at 4 °C. Figure 3B shows the optical and SEM images of the geometries and roughened surfaces formed by firmly inter-linked hydroxyapatite by gelatin of the printed G/H scaffold. The interconnected pores were well attained, and the geometries were similar with the designed structure (strut size: 393.1 ± 17.5 μm, pore size: 402.7 ± 26.3 μm, porosity: 82.3 ± 2.0%) (Figure 3C). To determine the HA concentration embedded in the G/H structure, we compared the HA weight fraction between the printing ink (before printing) and printed structure (after printing), and there was no significant difference in the HA concentration before and after printing (Figure 3D).

To confirm the composition in the G/H scaffolds, FT-IR and XRD tests were performed. Based on the absence of glycerol peaks, the remnant glycerol has been removed during the washing process (Figure 3E). Also, XRD showed that typical HA peaks were observed in the fabricated G/H scaffold, indicating that HA resided well in the scaffold (Figure 3F).

To assess the mechanical properties of the G/H scaffold (size = 4.5 × 4.5 × 3.0 mm^3^), several compressive stress-strain tests of the scaffold under wet-state were performed (Figure 3G). The recoverable phenomenon was clearly observed in the optical images (with 40% strain), indicating the G/H mesh structure steadily experienced the externally applied strain and rapidly recovered its initial form when the deformation pressure was eliminated. To quantify the recoverable properties of the G/H scaffold, various cycling tests until strain of 40% were performed, and the results showed that the G/H scaffold had good recovery properties without structural collapse, which is essential for efficient use of implantable scaffolds or injectable structures 43. However, a few hystereses during the compression/recovery cycles were observed, although the recoverable behavior remained unchanged for several cycles. A similar recoverable material phenomenon has been observed in several organic/inorganic biocomposite systems designed using leaching methods 44. It was shown that the recoverable behavior was due to the microporous structure, in which inorganic particles were strongly associated with highly rubbery polymers 45. Sequential loading-unloading compression tests on various pore sizes showed typical elastic recovery regardless of the pore sizes, but the maximum recoverable strain was higher with increased pore size (Figure S2). In addition, microstructure within the scaffolds also affects elastic recovery. Shah et al. prepared two different microstructures, “crater-web” and “monolithic”, of poly(ε-caprolactone) (PCL)/HA-based composites by controlling the evaporation rate of solvents. The monolithic structure with strongly inter-linked HA by PCL could absorb the external stresses and show the hyper-elastic properties, while the crater-web structure could not 9. Similar to previous works, we believe that the highly porous and inter-linked HA by gelatin within G/H microstructure displaying robust linkage between HA particles can induce an entropic response to the external strain of the microporous, eventually developing a stable recoverable behavior.

### Bioactivity of hPE and *in vitro* cellular activities of gelatin/HA/hPE scaffolds

Human placental extracts (hPE) have been used as potential therapeutic agents for wound healing because they contain various growth factors such as hepatocyte growth factor, epidermal growth factor, and transforming growth factor 46-49, and antioxidant and anti-inflammatory factors 50, 51. In addition to these growth factors and bioactive molecules, hPE contains polydeoxyribonucleotide, which is a potential stimulator of osteogenesis and angiogenesis, many enzymes, vitamins, and fatty acids (Table 1) 52-57. Because of the abundance of growth factors and other components of placental extracts, various researchers have attempted to apply them to cutaneous wound healing, facial nerve diseases, osteoarthritis models, etc. (Table 2).

**Figure 4:**
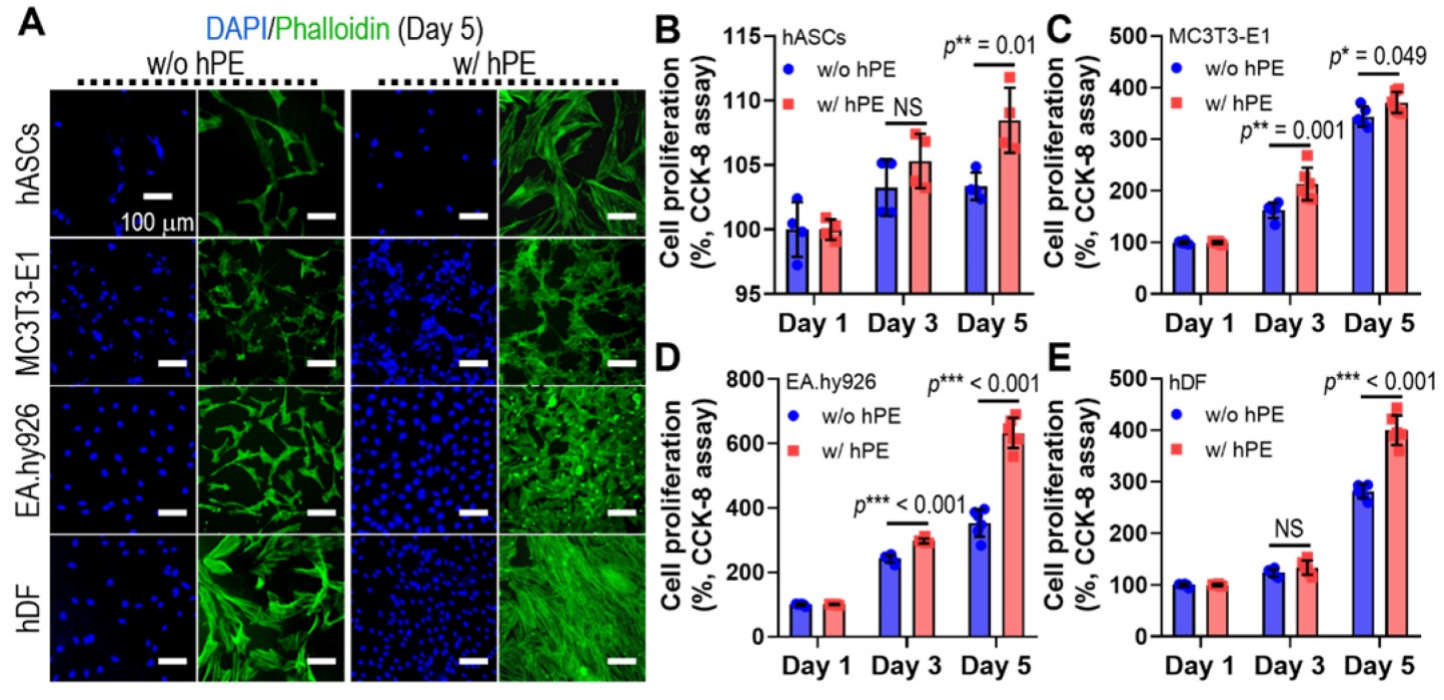
(A) DAPI (blue; cell nuclei) and phalloidin (F-actin; green) staining results after 5 days cell-culture; human adipose derived stem cells (hASCs), pre-osteoblast (MC3T3-E1), human endothelial cell line (EA.hy926), and human dermal fibroblast (hDFs) with and without hPE. (B) Cell proliferation, determined using CCK-8, for each cell type. (NS = not significant, ^*^p < 0.05, ^**^p < 0.005, ^***^p < 0.001)

To assess the effect of hPE on the activities of various cells (hASCs, MC3T3-E1, EA.hy926, and hDFs), we measured the DAPI/phalloidin stainning at day 5 (Figure 4A) and cell proliferation (Figure 4B-E), as determined using CCK-8 cell proliferation assay. hPE clearly enhanced the metabolic activities of the cultured cells, and even a high amount (2 mg/ml) of hPE did not exert any cytotoxic effect on the cells.

**Figure 5:**
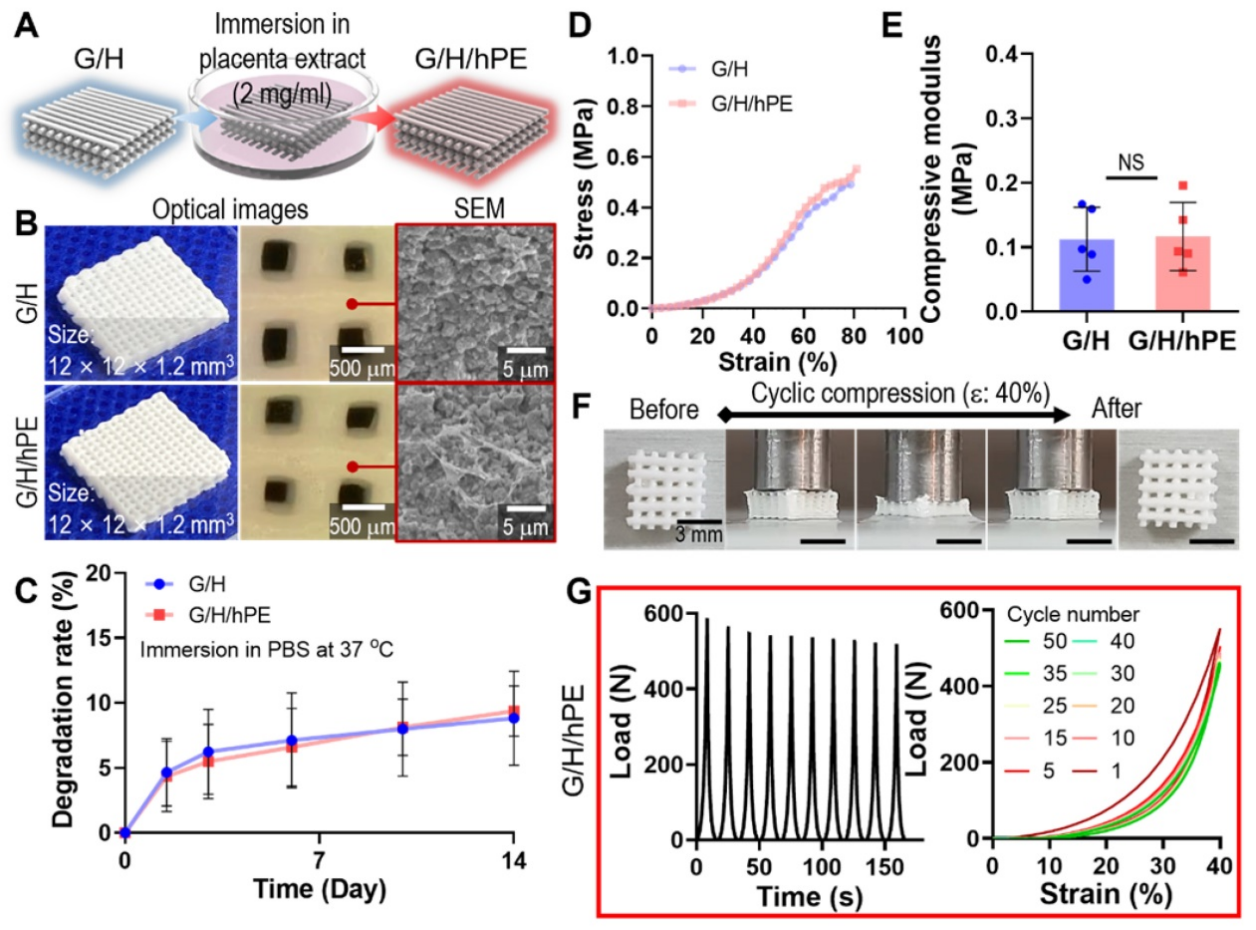
(A) A schematic of hPE-coating process on G/H scaffold (G/H/hPE), and (B) the optical/SEM images of fabricated G/H and G/H/hPE scaffolds. (C) The degradation rate of the G/H and G/H/hPE scaffolds in PBS at 37 °C. (D) Stress-strain curve and (E) the measured compressive modulus of the scaffolds. (F) Photograph series of one-cycle of compressive loading/unloading and (G) compressive load *vs.* times and compressive load *vs.* strain graph of G/H/hPE scaffolds. All the tests were performed in wet state.

Although gelatin and HA can be considered promising biomaterials for bone regeneration, further bioactive improvement of the scaffold is required to successfully achieve regeneration of the bone tissue. To attribute bioactive properties on the G/H scaffold, we coated the scaffold with 2 mg/ml of hPE by pipetting it onto the G/H scaffold and dried it, as shown in Figure 5A-B. After the hPE coating processes, there were insignificant differences on the degradation rate (G/H: 8.83 ± 3.61%, G/H/hPE: 9.39 ± 1.94% at day 14) (Figure 5C) and compressive modulus (G/H: 0.11 ± 0.05 MPa, G/H/hPE: 0.12 ± 0.05 MPa) (Figure 5D-E). In addition, the recoverable properties of the G/H/hPE scaffold were similar to G/H scaffold (Figure 3G, Figure 5F-G, and Movie 1). The elastic modulus of rat bone is 4.9 ~ 8 GPa 58, and cortical and cancellous bone in human is 12 ~ 18 GPa and 0.1 ~ 0.5 GPa, respectively 59. As such, G/H scaffolds fabricated in this study have a much lower modulus compared to natural bones. Similarly, previous reports have suggested that the main limitation of collagen or gelatin based hydroxyapatite is the low mechanical strength 8. Lin et al., have reported that the 3D printed collagen/hydroxyapatite has the range of 0.09 ~ 0.15 MPa in the elastic modulus 39, and Zhang et al., also reported that bulk gelatin/hydroxyapatite scaffolds have 0.2 MPa of maximum strength 60. In general, the bioceramic scaffolds have been reinforced by sintering processes 61. However, the natural polymer/bioceramic composite scaffolds cannot be sintered due to the natural polymer denaturation 62. Nevertheless, the G/H scaffold could provide the cell-adhesive motif compared to the pure bioceramic scaffolds 63. Considering the expected biological and elastic properties of G/H scaffolds, we believe that it could be adopted into the non-load bearing bone tissue regeneration.

**Figure 6:**
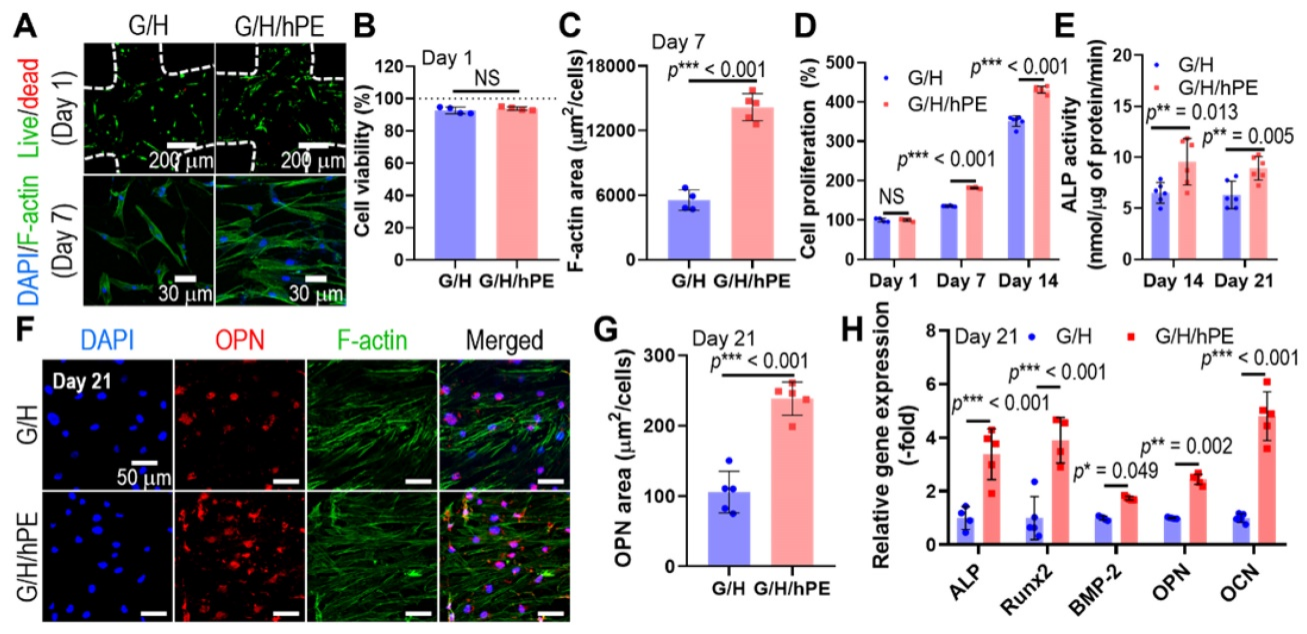
(A) Live (green)/dead (red) images after 1 day and DAPI (blue)/phalloidin (green) images of hASCs after 7 days in culture with the scaffolds. (B) Cell viability and (C) F-actin area. (D) Cell proliferation, determined using the CCK-8 assay, and (E) alkaline phosphatase (ALP) activities of the cells in the G/H and G/H/hPE scaffolds. (F) DAPI (blue)/phalloidin (green)/osteopontin (OPN; red) fluorescence images of the hASCs cultured on the scaffolds on day 21 and (G) measured OPN positive area. (H) Relative gene expression of *ALP, RUNX-2, BMP-2, OPN*, and *OCN* on day 21. (NS = not significant, ^*^p < 0.05, ^**^p < 0.005, ^***^p < 0.001)

Human adipose-derived stem cells (hASCs) have been widely used for the biological evaluation of various scaffolds due to their multi-potent differentiation including osteogenesis 64. Therefore, the osteogenic effects of the G/H and G/H/hPE scaffolds were assessed by the culture of the hASCs with the osteogenic differentiation medium 65. Toxicity and biocompatible properties of the fabricated G/H and G/H/hPE scaffolds were assessed using fluorescence images, live (green)/dead (red) at day 1 and DAPI (blue)/phalloidin (green) at day 7 (Figure 6A). As shown in the quantitative analysis of the fluorescence images (Figure 6B-C), appropriate cellular activities, such as cell viability and cell proliferation were observed over both G/H and G/H/hPE scaffolds indicating non-toxicity and biocompatibility. However, cell proliferation and F-actin area (%) of the hASCs cultured in the G/H/hPE scaffold were higher than those cultured in the G/H scaffold. By day 1, no significant difference was observed in the scaffolds; however, on days 7 and 14, a significantly higher proliferation of the cultured hASCs was detected in the G/H/hPE scaffold (G/H = 135.4 ± 1.1%, G/H/hPE = 181.5 ± 1.5% at day 7 and G/H = 351.1 ± 13.3%, G/H/hPE = 430.9 ± 8.7% at day 14) (Figure 6D). From these results, we can conclude that hPE induced meaningful metabolic activities. The effect of hPE on cell proliferation has also been evaluated by several researchers. According to Rameshbabu et al., the viability of human fibroblasts and keratinocytes was significantly enhanced by adding placental extracts to a nanofibrous scaffold 66. Han et al. reported that placental extract injection could improve the proliferation of osteoblast-like cells (MG-63) 67. This phenomenon could be due to various cell-supporting growth factors or anti-inflammatory components in placental extracts.

To observe the effect of placental extracts coated on the G/H scaffold on *in vitro* osteogenic activities, we measured ALP activity in hASCs cultured on the scaffolds for 14 and 21 days (Figure 6E). ALP activity in cells cultured on the G/H/hPE scaffold was significantly greater than that in cells cultured on the G/H scaffold in both time points (G/H = 6.4 ± 7.2 nM/μg of protein/min, G/H/hPE = 9.6 ± 2.3 nM/μg of protein/min at day 14 and G/H = 6.3 ± 1.3 nM/μg of protein/min, G/H/hPE = 8.9 ± 1.2 nM/μg of protein/min at day 21). In addition, osteopontin (OPN) expression by hASCs grown on the scaffolds was observed in the fluorescence images, indicating a trend similar to that of ALP activity (Figure 6F-G). In addition, expression of the osteogenic genes *ALP, Runx2, BMP2, OPN*, and *OCN* is shown in Figure 6H, and the primary and late osteogenic genes were significantly upregulated in the hASCs cultured on the G/H/hPE scaffold compared to the G/H scaffold (*ALP*: 3.4-fold, *Runx2*: 3.9-fold, *BMP2*: 1.7-fold, *OPN*: 2.4-fold, and *OCN*: 4.8-fold at day 21). Although the placental extract (PE) has been widely used as a therapeutic treatment, the osteogenic differentiation mechanism of PE remains to be unclear 31. Nevertheless, similar results proposing the osteogenic potential of placenta extract have been reported. Elevation of collagen type I synthesis and ALP activities of osteosarcoma cell line (Saos-2) by porcine placental extract were observed by Wang et al 31. Additionally, research on the effects of placental extract in ovariectomized (OVX) mice reported that placental extract could enhance bone formation in OVX mice and augment ALP activity in osteoblast-like cells 67. These results might be obtained by the synergistic effects of the various component in PE. Short-term treatment of transforming growth factor-beta (TGF-β), which is one of the growth factors that existed in PE, is known for chondrogenesis and osteogenesis 68. In addition, hepatocyte growth factor (HGF) also promotes the osteogenic differentiation of mesenchymal stem cells by rapid phosphorylation of p38 signaling 69. Therefore, we expected that the osteogenic activities of the scaffold would be significantly increased owing to various hPE bioactive components including TGF-β and HGF present on the 3D G/H scaffold.

### *In vivo* bone formation of hPE-supported G/H scaffold

**Figure 7:**
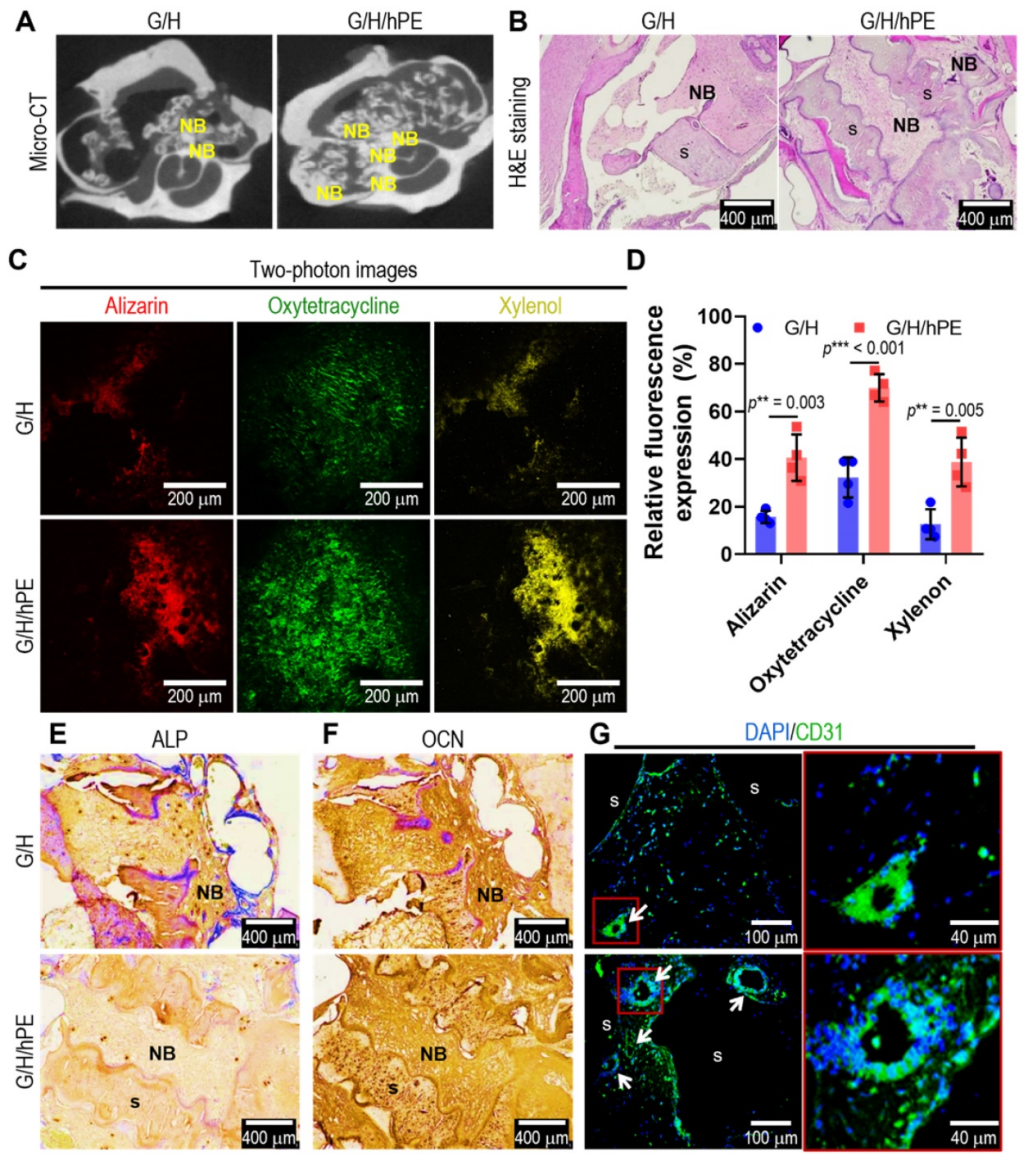
(A) Micro-CT images and (B) hematoxylin and eosin (H&E) staining after 12 weeks of the scaffold implantation. 'NB' and 's' indicating new bone formation and scaffold, respectively. (C) Two-photon fluorescence images of alizarin (red)/oxytetracycline (green)/xylenol (yellow) and (D) relative fluorescence expression (%). Immunohistochemistry images of (E) ALP and (F) OCN and immunofluorescence images of (G) DAPI (blue)/CD31 (green). Arrows indicating vascular formation. (NS = not significant, ^*^p < 0.05, ^**^p < 0.005, ^***^p < 0.001)

To assess bone formation in the scaffolds (G/H and G/H/hPE), an *in vivo* rat model of mastoid obliteration was used. Figure 7A shows micro-CT images demonstrating a higher osteogenesis area in the G/H/hPE scaffold than in the G/H scaffold (Hounsfield unit by image J; G/H group: 148.3 and G/H/hPE group: 163.0), but the difference was not statistically significant (*p* = 0.1175). In addition, the results of H&E staining showed much higher new bone formation, as shown by the NB marks, in the G/H/hPE scaffold than in the G/H scaffold (Figure 7B).

To examine the capability of the scaffolds to promote endochondral ossification and angiogenesis, alizarin (red)/oxytetracycline (green)/xylenol (yellow) was observed in two photon images (Figure 7C). Although newly formed and calcified bone tissue was observed in the G/H and G/H/hPE scaffolds, much greater bone formation, as indicated by alizarin/oxytetracycline/xylenol staining, was detected in the G/H/hPE scaffold than in the G/H scaffold (alizarin: G/H = 15.6 ± 2.7% and G/H/hPE = 40.6 ± 9.7% / oxytetracycline: G/H 32.28 ± 8.4% and G/H/hPE = 70.0 ± 5.7% / xylenol: G/H = 12.6 ± 6.4% and G/H/hPE = 38.8 ± 10.2%) (Figure 7D). The results were consistent with the histological results (ALP, OCN, CD31), and markedly higher levels of OCN and CD31 were detected in the G/H/hPE scaffold (Figure 7E-G). The results showed that the hPE bioactive components loaded in the gelatin/HA scaffold clearly induced high levels of angiogenesis and bone formation. Based on the in vivo results, we can conclude that the mechanically stable G/H scaffold loaded with hPE bioactive components can clearly enhance osteoinduction/osteoconduction, eventually accelerating bone formation in an *in vivo* model of mastoid obliteration.

## Conclusions

In this study, gelatin/HA scaffolds coated with human placental extract were fabricated using a 3D printing process. Through controlled printing conditions supplemented with the processing agent glycerol, stable printability was achieved for fabricating the scaffold. The fabricated G/H/hPE scaffold presented outstanding elastic and recoverable physical properties and showed significantly enhanced biocompatibility and osteogenic activities as determined using human adipose stem cells compared to G/H scaffolds. Furthermore, *in vivo* results using a rat mastoid obliteration model demonstrated outstanding bone formation, showing the regenerative effect of the G/H/hPE scaffold compared with the gelatin/HA scaffold. The effects were clearly due to the various bioactive components (growth factors, cytokines, proteins, minerals, etc.) of placental extracts laden on the scaffold. Based on *in vitro* and *in vivo* studies, the combination of hPE and gelatin/HA scaffold has great potential for use as a biomedical material for regenerating bone tissue.

## Methods

### Materials

Gelatin type A (Mw: 50,000; MP Biomedicals Korea, South Korea), hydroxyapatite (particle size: 54.7 nm, HA; Sukgyung AT, South Korea), and glycerol (Sigma-Aldrich, St. Louis, MO, USA) were used for the preparation of composite printing ink. 1-Ethyl-3-(3-dimethylaminopropyl) carbodiimide (EDC), N-hydroxysuccinimide (NHS), β-glycerophosphate, ascorbate-2-phosphate, dexamethasone, Triton X-100, and paraformaldehyde were purchased from Sigma-Aldrich. Human adipose-derived stem cells (hASCs), pre-osteoblast cells (MC3T3-E1), human endothelial cells (EA.hy926), and human dermal fibroblasts (hDFs) were purchased from ATCC (Manassas, VA, USA). Placental extract was kindly donated by Prof. J. H. Choi and G. W. Cho at Chosun University.

### Preparation of gelatin/HA ink

For the preparation of printing solution containing gelatin/HA with glycerol (G/H w/ gly), 1 g of gelatin type A powder (MP Biomedicals, South Korea) and 2.33 g of hydroxyapatite powder (Sukgyung AT) were dissolved in 10 ml of glycerol solution (30 v/v%). For the preparation of printing solution containing gelatin/HA, equal amounts of gelatin and hydroxyapatite were dissolved in 10 ml of 3^rd^ distilled water instead of a 30 v/v% glycerol solution (G/H w/o gly).

### Rheological measurement

The complex viscosities of gelatin/HA solutions with and without 30 v/v% glycerol were measured. A rotational rheometer (Bohlin Gemini HR Nano; Malvern Instruments, Surrey, UK) equipped with a cone-and-plate (4° cone angle, 40 mm diameter, and 150 μm gap) was used, and temperature sweep was conducted (15 °C - 40 °C, ramping speed of 2 °C/min) at 1 Hz frequency and 1% strain.

### Single line test for the gelatin/HA with and without glycerol

A 3D printer (DTR2-2210T-SG; Dongbu Robot, South Korea) equipped with a heating barrel and a working plate was used to evaluate the printability of G/H with and without glycerol. For the line extrusion tests, a 400 μm printing nozzle was used, and the pneumatic pressure was set to 30 kPa. The diameter of the printed struts was measured using FiJi software.

### Scaffold fabrication

Gelatin/HA inks with and without glycerol were printed with a nozzle of 250 µm under the processing conditions (barrel and printing stage temperatures: 33-34 °C and 5 °C, respectively; nozzle moving speed: 10 mm/s; pneumatic pressure: 90-110 kPa). The fabricated structures were cross-linked with 100 mM EDC/NHS solution in 80% ethanol solution for 24 h at 4 °C. Then, the constructs were washed with 3^rd^ distilled water and 100% ethanol for five times. The samples were lyophilized and stored at 4 °C. To coat the structures with hPEs, they were immersed in hPE solution (2 mg/ml) for 30 min and then lyophilized again.

### Characterization of the printed constructs

The weight of the lyophilized and non-crosslinked G/H scaffolds was measured (WG/H) to assess the HA concentration in G/H with glycerol before and after 3D printing. The gelatin in the G/H scaffold was removed with 3^rd^ distilled water, and the remnant was centrifuged (12000 rpm for 5 min). The procedure for gelatin removal and centrifugation was repeated 20 times, and the remaining HA was measured (W_HA_). Through the weights, the HA concentration was calculated using the following equation, W_HA_/W_G/H_ × 100.

The surface and cross-sectional morphologies of the fabricated scaffolds were observed using scanning electron microscopy (SEM, SNE-3000M, SEC Inc., South Korea). Based on the optical images, the pore and strut sizes of the printed structures were measured using FiJi software (National Institutes of Health, Bethesda, MD, USA).

The porosity (%) of the scaffolds was calculated using the following equation: [1 - (M × x_gel_/ρ_gel_ + M × x_HA_/ρ_HA_)/V_total_] × 100, where M, x, ρ, and V indicate the weight, weight fraction, density, and volume of the scaffolds (assumed cuboid), respectively.

To observe the HA crystal planes, X-ray diffraction (XRD) was performed using a WAXD (X'PertPRO MPD, PANalytical, Netherlands) with CuK_α_ radiation under beam conditions of 40 kV and 20 mA with the collection of a spectrum at 2θ = 15-45°.

The chemical components of the fabricated scaffolds were assessed by Fourier transform infrared (FT-IR) spectrometer (model 6700, Nicolet, West Point, PA). The IR spectra represented the mean of 30 scans at 600 - 4000 cm^-1^.

The cuboid geometry (4.5 × 4.5 × 3.0 mm^3^) of G/H scaffolds was subjected to cyclic compression tests. Before the tests, the samples were immersed in PBS for 1 h. Then, the samples were exerted 40% strain for 50 cycles (30 mm/min) using a universal testing machine (UTM; Top-tech, South Korea). The compressive stress-strain curves were assessed and modulus was calculated at the linear region in the stress-strain curves.

The cuboid geometry (4.5 × 4.5 × 3.0 mm^3^) of G/H and G/H/hPE scaffold were immersed in PBS at 37 °C to assess the degradation rate. For each time point, the scaffolds were washed with 3^rd^ distilled water and lyophilized to measure the remained weight. Through the weights, the degradation weight was calculated using the following equation, [1- W_t_/W_i_] × 100, where W_i_ and W_t_ indicate the initial weight and weight of scaffolds at each time point, respectively.

### *In vitro* test

Human adipose-derived stem cells (hASCs; ATCC), MC3T3-E1 cells (ATCC), EA.hy926 cells (ATCC), and human dermal fibroblasts (hDFs, ATCC) were used to evaluate the effects of hPE. Dulbecco's modified Eagle's medium-low glucose (DMEM-lg; HyClone, USA) supplemented with 10% of fetal bovine serum (FBS, Gemini Bio-Products) and 1% penicillin-streptomycin (HyClone) was used for hASCs. Dulbecco's modified Eagle's medium-high glucose (DMEM-hg; HyClone) containing 10% FBS and 1% PS was used for EA.hy926 cells and hDFs cells, and alpha-minimum essential medium (Gibco) with 10% FBS and 1% PS was used for MC3T3-E1 cell culture.

Each cell type (hASCs, MC3T3-E1, EA.hy926, and hDFs) was seeded onto 24 well cell culture plates (1 × 10^4^ cells/well) for 5 days. All growth media were mixed with hPE (2 mg/ml), and growth medium without hPE was used as a control. The cell culture medium was changed every two days.

Cell proliferation was assessed using the Cell Counting kit-8 (CCK-8 assay, Dojindo, Japan) following the manufacturer's protocol. Briefly, 180 μl of growth medium and 20 μl of the CCK-8 assay solution were pipetted onto the samples. After 30 min of incubation at 37 °C, absorbance was measured using a microplate reader (EL800, Bio-Tek) at 450 nm.

To visualize the morphology of the cells cultured with or without hPE, the cells were fixed with 3.7% formaldehyde (Sigma-Aldrich) after 5 days of culture. After permeabilization with 0.1% Triton X-100 (Sigma-Aldrich), the cells were stained with diamidino-2-phenylinodole (DAPI, 1:100 dilution in PBS; Invitrogen, USA) and FITC phalloidin (1:100 dilution in PBS; Invitrogen) for 45 min. The stained cells were observed under a confocal microscope (LSM700, Carl Zeiss, Germany).

To evaluate effect of gelatin/HA composite scaffolds with or without hPE, 5 × 10^4^ hASCs were seeded onto sterilized G/H and G/H/hPE scaffolds. Growth medium was mixed with 100 μM dexamethasone (Sigma-Aldrich), 10 mM β-glycerophosphate (Sigma-Aldrich), and 50 μM ascorbate-2-phosphate (Sigma-Aldrich) and used as osteogenic differentiation medium. After 7 days of cell culture in growth media, osteogenic medium was used to induce osteogenesis in hASCs. The medium was changed every two days.

To determine cell viability, cells cultured on the scaffolds were stained using a live/dead assay. Briefly, 0.15 mM calcein AM and 2 mM ethidium homodimer-1 (Thermo Fisher Scientific, USA) were added at 37 °C for 30 min. Fluorescence images were captured using a confocal microscope (LSM 700), and cell viability was measured using FiJi software based on the fluorescence images.

To examine osteopontin (OPN) expression in the cells after 21 days of cell culture, the cell-seeded scaffolds were fixed with 3.7% formaldehyde. Then, permeabilization was performed using 2% Triton X-100 (Sigma-Aldrich) for 1 h. To prevent non-specific binding, the samples were immersed in 2% bovine serum albumin (BSA). Rabbit monoclonal anti-osteopontin antibody (1:100; Abcam, UK) was added to the samples overnight at 4 °C, and the samples were incubated with Alexa Fluor 594-conjugated goat anti-rabbit secondary antibody (1:200; Invitrogen) for 1 h at room temperature. Cell nuclei and F-actin were counterstained with DAPI (Invitrogen; 1:100 dilution) and FITC phalloidin (Invitrogen; 1:100 dilution). A confocal laser-scanning microscope (LSM-700, Zeiss, Germany) was used to visualize the prepared samples. The OPN-positive area was measured using FiJi software.

A real-time polymerase chain reaction (RT-PCR) was performed using RNA isolated fromhASCs cultured on the scaffolds for 21 days to measure the relative gene expression levels of alkaline phosphatase (ALP), runt-related transcription factor 2 (Runx2), bone morphogenetic protein 2 (BMP2), osteopontin (OPN) and osteocalcin (OCN). The TRIzol reagent (Sigma-Aldrich) was used for RNA isolation. The purity and concentration of the extracted RNA were measured using a spectrophotometer (Optizen Pop; K Lab, Daejeon, South Korea). RNA (500 ng) was used for cDNA synthesis using ReverTra Ace qPCR RT Master Mix (Toyobo, Japan). RT-PCR was performed using the StepOnePlus Real-Time PCR system (Applied Biosystems, Foster City, USA) and THUNDERBIRD SYBR qPCR Mix (Toyobo), according to the manufacturer's protocol. The gene specific primers were used as follows: ALP (forward, ggc acc tgc ctt act aac tcc; reverse, gtg ggt ctc tcc gtc cag), runt-related transcription factor 2 (Runx-2; forward, cag tga cac cat gtc agc aa; reverse, gct cac gtc gct cat ttt g), bone morphogenic protein 2 (BMP-2; forward, cag acc acc ggt tgg aga; reverse, cca ctc gtt tct ggt agt tct tc), osteopontin (OPN; forward, aag ttt cgc aga cct gac atc; reverse, ggg ctg tcc caa tca gaa gg), osteocalcin (OCN; forward, tga gag ccc tca cac tcc tc-30; reverse, acc ttt gct gga ctc tgc ac), and b-actin (Actb; forward, tcc aaa tat gag atg cgt tgt t; reverse, tgc tat cac ctc ccc tgt gt).

### Surgical procedure for rat mastoid obliteration

Twelve healthy Sprague-Dawley rats weighing 200-250 g (Samtakobio, South Korea) were used in this study. The animal experiments were approved by the Institutional Animal Care and Use Committee of Chonnam National University Medical School (Permit Number: CNU CIACUC2019-S0001). For rat mastoid obliteration, gelatin/HA scaffolds (2 × 2 × 2 mm^3^, 3.54 ± 0.24 mg) with 100 μl of PBS (G/H, control group) and gelatin/HA scaffolds with hPE (100 μl, 2 mg/ml) (G/H/hPE, experimental group) were prepared. The dose of hPE was selected based on the other research 31, 70. Clean surgical drapes are employed in the infra-auricular area to provide a physical barrier that protects the surgical field from contamination after antiseptic painting using a povidone after shaving the hairs around the auricle using an electric clipper. Under inhalation anesthesia using isoflurane, dental lidocaine (1:100000) was administered around the auricle for hemostasis. After a skin incision was made around the auricle, a subcutaneous dissection was performed to identify the right bulla. The hole in the bulla was made using a portable mini-hand drill and irrigated using suction to clean out bone dust, which may interfere with bone formation. The bulla were obliterated using G/H (n = 6) and G/H/hPE (n = 6). The skin incision site was approximated using an autoclip (Jeung Do Bio & Plant Co., Seoul, South Korea) after hemostasis was achieved using a portable cautery pen set (Sound Co. Rowville, Australia).

### Fluorescent labelling

During the 12 weeks healing period, fluorescent bone markers were injected 55 to observe the dynamics of new bone formation. Three weeks after the operation, 20 mg alizarin red S/kg of body weight was administered intraperitoneally (IP) (Sigma-Aldrich), 20 mg oxytetracycline HCl/kg of body weight (Sigma-Aldrich) was injected IP at 6 weeks post-surgery, and 20 mg xylenol/kg of body weight (Sigma-Aldrich) was injected IP at 9 weeks post-surgery. All dyes were prepared immediately before use in saline.

### *Ex vivo* micro-CT evaluation

The animals were sacrificed at 12 weeks post-surgery. After extraction of bullae, they were fixed with 10% formalin. To evaluate new bone formation, *ex vivo* micro-CT was performed using a Quantum GX micro-CT imaging system (PerkinElmer, Hopkinton, MA, USA) at the Korea Basic Science Institute (Gwangju, Korea). The setting conditions were similar to those used in our previous report 71. Briefly, the condition was set at 90 kV/80 µA with a field of view of 45 mm (voxel size, 90 µm; scan mode, high resolution; scan time, 4 min), and an approximate dose (X-ray) of 162 mGy radiation per scan. To quantify and objectively compare the amount of mineralization by osteogenesis, we cropped the obliterated bulla from the axial micro-CT images and visualized the radiopaque portion of each slice. From the axial micro-CT image, we measured the mean grey values (Hounsfield units) using ImageJ 1.52a (Wayne Rasband, National Institute of Health, USA).

### Fluorescence imaging evaluation by multiphoton microscopy

To image bone regeneration, we used two-photon fluorescence microscopy (TPM) with an intravital multiphoton microscope (SP8-MP; Leica, Wetzla) at the Korea Basic Science Institute (KBSI, Gwangju, Korea). Bone samples were stored in PBS for TPM imaging. The fluorophores were irradiated using the InSight DS Plus laser system (Spectra-Physics, Santa Clara, CA, USA) at an excitation wavelength of 800 nm (xylenol), 1040 nm (oxytetracycline), and 1180 nm (alizarin). Emissions were collected at 365-490 nm (oxytetracycline), 375-570 nm (xylenol), and 538-580 nm (alizarin).

### Histopathological evaluation

After *ex vivo* micro-CT and multiphoton microscopy imaging, all bullae were subjected to decalcification and serial dehydration, followed by paraffin embedding. The sections were stained using hematoxylin-eosin (HE), immunohistochemistry for OCN and ALP, and immunofluorescence for CD31. Tissue sections were deparaffinised using routine protocols. The endogenous peroxidase activity of the tissue was blocked following incubation with 0.3% (*v*/*v*) hydrogen peroxide for 20 min. This was followed by blocking of nonspecific reactions for 1 h using 5% (*v*/*v*) NGS (Vector ABC Elite Kit; Vector Laboratories, Burlingame, CA, USA) in 0.3% (*v*/*v*) Triton X-100. The sections were subsequently incubated with the following primary antibodies at 4 °C overnight: anti-ALP antibody (Abcam, ab108337, diluted 1:250) and anti-OCN antibody (Abcam, ab13420, diluted 1:250). Biotinylated goat anti-rabbit immunoglobulin G (IgG) (Vector ABC Elite Kit; Vector Laboratories) was incubated at room temperature for 1 h to detect primary antibody binding. Immunoreactivity was measured using an avidin-biotin peroxidase complex (Vector ABC Elite Kit; Vector Laboratories), which was incubated for 1 h at room temperature according to the manufacturer's instructions. The peroxidase reaction was carried out with a diaminobenzidine substrate (contained in the DAB kit; Vector Laboratories) assay, according to the manufacturer's instructions. For immunofluorescence, sections were treated with rabbit monoclonal anti-CD31 antibodies (Abcam, diluted 1:100) at 4 °C. The samples were then incubated with Alexa Fluor 488-conjugated goat anti-rabbit secondary antibody (1:200; Invitrogen) for 1 h at room temperature and counterstained with DAPI (1:100; Invitrogen). Fluorescence images were captured using a confocal microscope (LSM700, Zeiss).

### Statistical analysis

All data are presented as mean ± standard deviation (SD). The SPSS software (ver. 20.0, SPSS Inc., Chicago, IL, USA) was used for statistical analysis. Student's *t*-test was performed for comparisons between the two groups. Values of p^*^ < 0.05, p^**^ < 0.005, and p^***^ < 0.001 were considered statistically significant.

## Figures and Tables

**Table 1:** Components of placental extracts.

Types	Name	Ref.
*Growth factors*	Hepatocyte growth factor	47
	Epidermal growth factor	48
	Transforming growth factor	49
	Insulin-like growth factor-1	72
	Vascular endothelial growth factor	72
	Fibroblast growth factor	73
	Keratinocyte growth factor	73
*Cytokines*	Relaxin	74
	TNF-α	75
	Leptin	76
*Proteins*	L-tryptophan	50
	Uracil	77
	Tyrosine	77
	Phenylalanine	77
*Other* *components*	Polydeoxyribonucleotide	78
	Adrenocorticotropic hormone	72
	Glycosphingolipids	73

**Table 2:** The application of placental extracts in tissue engineering.

Author	Doses	Experiment	Results	Ref.
Han et al.	0.1 - 10 μg/ml	*- In vitro* test Human osteoblast-like cells (MG63) and human breast cancer cell line (MCF-7) - *In vivo* test Oral administration to ovariectomized mice model (every day for 8 weeks)	- *In vitro* test Increased cell proliferation Improved estrogenic and ostoblastic activity - *In vivo* test 17β-estradiol and ALP activities increased Improvement of bone mineral density	67
Park et al.	0.02 ml	- *In vivo* test Subcutaneous injection into the full thickness cutaneous wound model (4 mm) on diabetic mice (every other day for 2 weeks)	Faster wound closure rates Reduced inflammatory Increased fibroblast growth factor 2 (FGF-2) expression and collagen synthesis	79
Hong et al.	2 μl	- *In vivo* test Injection into boundaries of the full thieckness skin wound defects (8 mm)	Decrease of wound size Increased TGF-β and VEGF expression	80
Jo et al.	0.1 - 0.2 cc	- Clinical application (32 facial spasm patients) Injection 1~2 cm deep, once a day, three times a week treated with acupuncture	72% of patients reported excellent improvement (grade 0, no symptom) 18% of patients improved grade 1 (increased blinking caused by external stimuli) 3% of patients improved grade 2 (mild noticeable fluttering)	81
Kim et al.	- 1.8 μl - 360 μl/ml for *in vitro* test - 0.022 - 0.4 ml/kg for *in vivo* test	*- In vitro* test Human osteoblast-like cells (MG63) Glycosaminoglycan degrdataion analysis of rabbit knee articular cartilage explants in culture media - *In vivo* test Osteoarthritis rat (every day for 2 weeks)	*- In vitro* test Inhibition of matrix metalloproteinase (MMP)-2 activity of MG-63 cells Inhibition of proteoglycan degradation in rabbit knee articular cartilage explants - *In vivo* test MMP-2, -9 activities reduction in the cartilage	70
