# Supplementary material for: Highly elastic 3D-printed gelatin/HA/placental-extract scaffolds for bone tissue engineering
Source: Theranostics. 2022 May 13;12(9):4051–66. doi: 10.7150/thno.73146 (PMC9169369; doi:10.7150/thno.73146)
Supplement: Supplementary file 1 — Supplementary figures and movie legend. [file thnov12p4051s1.pdf]

Supplementary Materials

Highly elastic 3D-printed gelatin/HA/placental-extract scaffolds for bone tissue engineering

JiUn Lee,[a] Dongyun Kim,[a] Chul Ho Jang,[b,*] Geun Hyung Kim[a,c,*]

[a] Department of Biomechatronic Engineering, College of Biotechnology and Bioengineering, Sungkyunkwan University (SKKU), 16419, Suwon, Republic of Korea

[b] Department of Otolaryngology, Chonnam National University Medical School, Gwangju 61186, Republic of Korea

[c] Biomedical Institute for Convergence at SKKU (BICS), Sungkyunkwan University, Suwon 16419, Republic of Korea

Supplementary Figures & Movie

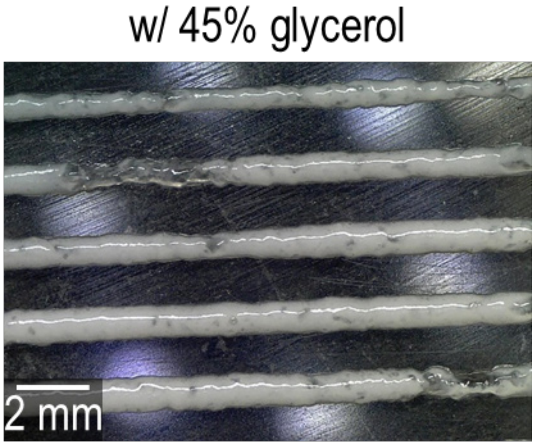

Figure S1. Single line extrusion tests of G/H solution dissolved in 45% glycerol.

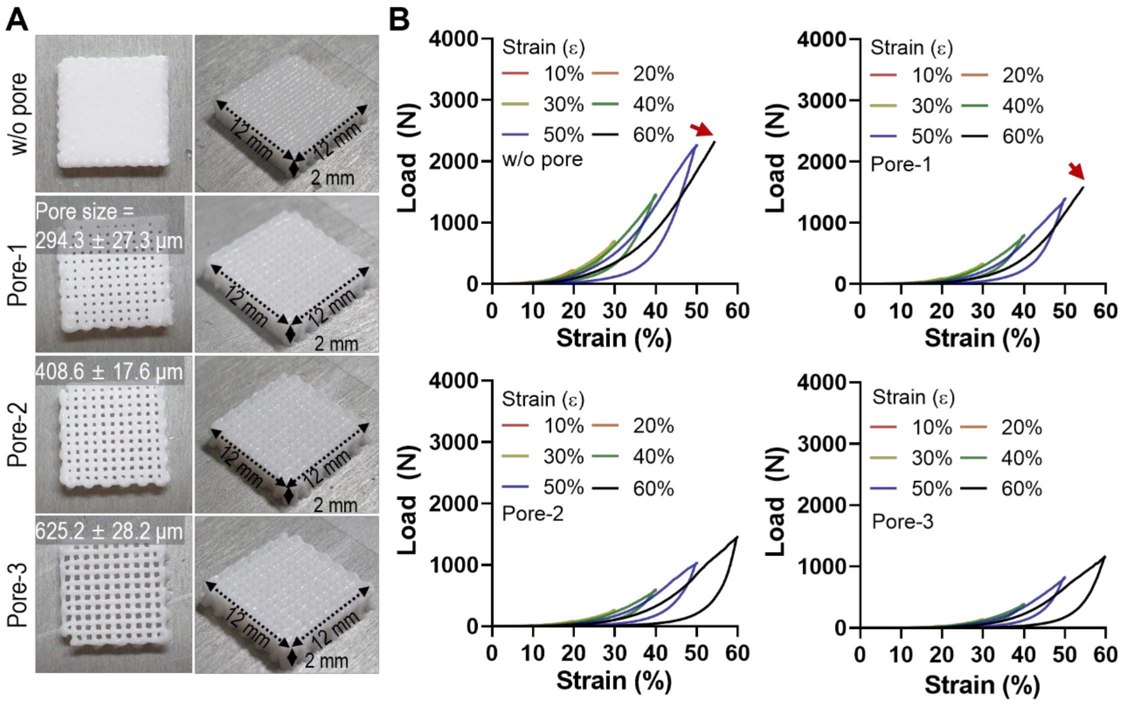

Figure S2. (A) Optical images of the G/H scaffolds with various pore sizes. (B) Sequential loading-unloading compression tests of G/H scaffolds under various strains (ε = 10 ~ 60%). Red arrows indicate structure fractures.

Movie 1. Cyclic compression test of G/H and G/H/hPE scaffolds.
